# Supplementary material for: Landscape of Genomic Alterations and PD-L1 Expression in Early-Stage Non-Small-Cell Lung Cancer (NSCLC)—A Single Center, Retrospective Observational Study
Source: Int J Mol Sci. 2022 Oct 19;23(20):12511. doi: 10.3390/ijms232012511 (PMC9604339; doi:10.3390/ijms232012511)
Supplement: Supplementary file 1 [file ijms-23-12511-s001.zip › Supplementary Table S1_List of EGFR mutations in pulmonary adenocarcinoma.pdf]

List of EGFR mutations in lung adenocarcinoma (ACA)

Exon18

EGFR:p.Glu709Ala(NM_005228):c.2126A>C: exon18)

EGFR:p.Glu709Lys(NM_005228):c.2125G>A: exon18)

EGFR:p.Gly719Ala(NM_005228):c.2156G>C: exon18)

EGFR:p.Gly719Ser(NM_005228):c.2155G>A: exon18)

EGFR:p.Gly719Cys(NM_005228):c.2155G>T: exon18)

EGFR:p.Lys714Asn(NM_005228):c.2142G>C: exon18) VUS

EGFR:p.Glu709_Thr710delinsAsp (NM_005228):c.2127_2129del: exon18)

Exon19

EGFR:p.Glu746_Ala750delGluLeuArgGluAla(NM_005228):c.2235_2249del:(exon19)

EGFR:p.Glu746_Ser752delGluLeuArgGluAlaThrSerinsVal(NM_005228):c.2237_2255T: exon19)

EGFR:p.Glu746_Leu747delinsValPro(NM_005228):c.2237_2240AATT>TTCC: exon19)

EGFR:p.Glu746_Pro753delGluLeuArgGluAlaThrSerProinsValAla(NM_005228):c.2236_2259GAATTAAGAGAAGCAACATCTCCG>GTAGCG: exon19)

EGFR:p.Glu746_Pro753delGluLeuArgGluAlaThrSerProinsValSer(NM_005228):c.2236_2259GAATTAAGAGAAGCAACATCTCCG>GTCTCG: exon19)

EGFR:p.Glu746_Ser752delGluLeuArgGluAlaThrSerinsVal(NM_005228):c.2237_2255T: exon19)

EGFR:p.Glu746_Thr751delGluLeuArgGluAlaThrinsAla(NM_005228):c.2236_2253GAATTAAGAGAAGCAACA>GCA: exon19)

EGFR:p.Glu746_Thr751delGluLeuArgGluAlaThrinsIle(NM_005228):c.2235_2252GGAATTAAGAGAAGCAAC>AAT: exon19)

EGFR:p.Leu747_Ala750delLeuArgGluAlainsPro(NM_005228):c.2239_2248C: exon19)

EGFR:p.Leu747_Pro753delinsSer(NM_005228):c.2240_2257del: exon19)

EGFR:p.Leu747_Ser752del(NM_005228):c.2239_2256del: exon19)

EGFR:p.Leu747_Thr751delLeuArgGluAlaThr(NM_005228):c.2238_2252del: exon19)

EGFR:p.Leu747_Thr751delLeuArgGluAlaThrinsPro(NM_005228):c.2239_2251C: exon19)

EGFR:p.Leu747_Ala755delLeuArgGluAlaThrSerProLysAlainsSerProGlnGly(NM_005228):c.2240_2264TAAGAGAAGCAACATCTCCGAAAGC>CTCCGCAAGG: exon19)

EGFR:p.Lys739_Ile744dup(NM_005228):c.2215_2232dup: exon19)

EGFR:p.Leu747Pro(NM_005228):c.2239_2240TT>CC: exon19)

EGFR:p.Lys745Asn(NM_005228):c.2235G>C: exon19)

EGFR:p.Glu746_Leu747delinsIlePro(NM_005228):c.2235_2241GGAATTA>AATTCCT: exon19)VUS

EGFR:p.Asp761Tyr(NM_005228):c.2281G>T: exon19) VUS

Exon20

EGFR:p.Ala767_Val769dupASV:c.2297_2305dup: exon20)

EGFR:p.Ser768_Asp770dupSerValAsp(NM_005228):c.55249004_55249012insAGCGTGGAC: exon20)

EGFR:p.Asp770_His773dupAspAsnProHis(NM_005228):c.2308_2316insACAACCCCC: exon20)

EGFR:p.His773_Val774dupHV(NM_005228):c.2316_2321dup: exon20)

EGFR:p.Pro772insHisAla(NM_005228):c.2314_2315insCCCACG: exon20)

EGFR:p.Ser768_Val769delinsIleLeu(NM_005228):c.2303_2305TCT: exon20)

EGFR:p.Ser768Ile(NM_005228):c.2303G>T: exon20)

EGFR:p.Cys781Phe(NM_005228):c.2342G>T: exon20),VUS

Exon21

EGFR:p.Leu858Arg(NM_005228):c.2573T>G: exon21)

EGFR:p.Leu861Gln(NM_005228):c.2582T>A: exon21)

EGFR:p.Ala864Pro(NM_005228):c.2590G>C: exon21) VUS

Compound mutations early stages

EGFR:p.Gly719Ala(NM_005228):c.2156G>C: exon18)	EGFR:p.Asp761Tyr(NM_005228):c.2281G>T: exon19)	TP53:p.Cys242Phe(NM_000546):c.725G>T: ex7)	
EGFR:p.Glu709Ala(NM_005228):c.2126A>C: exon18)	EGFR:p.Leu858Arg(NM_005228):c.2573T>G: exon21)	TP53:p.Gly199Leu(NM_000546):c.595_596GG>TT: ex6)	
EGFR:p.Ser768Ile(NM_005228):c.2303G>T: exon20)	EGFR:p.Leu858Arg(NM_005228):c.2573T>G: exon21)		
EGFR:p.Gly719Ser(NM_005228):c.2155G>A: exon18)	EGFR:p.Ser768Ile(NM_005228):c.2303G>T: exon20)	STK11:p.Asp327fs*9(NM_000455):c.980delA: ex8)	DDR2:p.Met657Arg(NM_006182):c.1970T>G: ex15
EGFR:p.Gly719Ala(NM_005228):c.2156G>C: exon18)	EGFR:p.Ser768Ile(NM_005228):c.2303G>T: exon20)		
EGFR:p.Glu709Ala(NM_005228):c.2126A>C: exon18)	EGFR:p.Gly719Ala(NM_005228):c.2156G>C: exon18)	TP53:p.Arg248Gln(NM_000546):c.743G>A: ex7)	

Compound mutations advanced stages

EGFR:p.Leu858Arg(NM_005228):c.2573T>G: exon21)	EGFR:p.Ala864Pro(NM_005228):c.2590G>C: exon21)		
EGFR:p.Glu709Ala(NM_005228):c.2126A>C: exon18)	EGFR:p.Gly719Ser(NM_005228):c.2155G>A: exon18)	TP53:p.Glu286Lys(NM_000546):c.856G>A: ex8)	
EGFR:p.Glu709Ala(NM_005228):c.2126A>C: exon18)	EGFR:p.Leu858Arg(NM_005228):c.2573T>G: exon21)		
EGFR:p.Gly719Cys(NM_005228):c.2155G>T: exon18)	EGFR:p.Ser768Ile(NM_005228):c.2303G>T: exon20)	TP53:p.Arg273Leu(NM_000546):c.818G>T: ex8)	
EGFR:p.Gly719Ala(NM_005228):c.2156G>C: exon18)	EGFR:p.Leu861Gln(NM_005228):c.2582T>A: exon21)		
EGFR:p.Gly719Cys(NM_005228):c.2155G>T: exon18)	EGFR:p.Ser768Ile(NM_005228):c.2303G>T: exon20)		
EGFR:p.Gly719Ala(NM_005228):c.2156G>C: exon18)	EGFR:p.Leu861Gln(NM_005228):c.2582T>A: exon21)		
EGFR:p.Glu709Lys(NM_005228):c.2125G>A: exon18)	EGFR:p.Lys714Asn(NM_005228):c.2142G>C: exon18)	EGFR:p.Gly719Ala(NM_005228):c.2156G>C: ex18)	FGFR3:p.Lys705Arg(NM_000142):c.2114A>G: ex16
EGFR:p.Lys739_Ile744dup(NM_005228):c.2215_2232dup: ex19)	EGFR:p.Lys745Asn(NM_005228):c.2235G>C: exon19)	CTNNB1:p.Ser45Phe(NM_001904):c.134C>T: ex3)	

	Early stages I-IIIA		Advanced stages IIIB-IV		*p*-value
	**n**	**%**	**n**	**%**	
**Exon 18**	**9**	**11,5**	**11**	**12,5**	1
**Exon 19**	**34**	**43,6**	**36**	**40,9**	0,84805325
EX19del	33		33		0,63634583
**Exon 20**	**11**	**14,1**	**9**	**10,2**	0,59843415
EX20ins	7		7		1
**Exon 21**	**24**	**30,8**	**32**	**36,4**	0,55090445
L858R	**21**		**24**		1
**All EGFR mutations**	78		88		0,97854265
**Patients EGFR+**	**72**		**78**		0,77769783
ACA all	567		650		0,98302673
Compound mutations	6	8,3	9	11,5	0,79917118
