# Supplementary material for: Landscape of Genomic Alterations and PD-L1 Expression in Early-Stage Non-Small-Cell Lung Cancer (NSCLC)—A Single Center, Retrospective Observational Study
Source: Int J Mol Sci. 2022 Oct 19;23(20):12511. doi: 10.3390/ijms232012511 (PMC9604339; doi:10.3390/ijms232012511)
Supplement: Supplementary file 1 [file ijms-23-12511-s001.zip › Supplementary Table S2_List of KRAS mutations in pulmonary adenocarcinoma.pdf]

KRAS mutations

	n all ACA	G12A	G12C	G12D	G12F	G12I	G12R	G12S	G12V	G13C
Early Stages I-IIIA										
n	229	17.0	98.0	34.0	3.0	0.0	1.0	4.0	43.0	11.0
%	100.0	7.4	42.8	14.8	1.3	0.0	0.4	1.7	18.8	4.8
Advanced Stages IIIB-IV										
n	260	11.0	102.0	36.0	5.0	1.0	3.0	7.0	60.0	7.0
%	100.0	4.2	39.2	13.8	1.9	0.4	1.2	2.7	23.1	2.7

	G13D	G13R	Q61H	Q61L	L19F	A146T	T148R	A59E	A59G
Early Stages I-IIIA									
n	5.0	1.0	6.0	2.0	1.0	1.0	1.0	1.0	1.0
%	2.2	0.4	2.6	0.9	0.4	0.4	0.4	0.4	0.4
Advanced Stages IIIB-IV									
n	7.0	0.0	19.0	4.0	2.0	0.0	0.0	0.0	0
%	2.7	0.0	7.3	1.5	0.8	0.0	0.0	0.0	0.0

Early Stages I-IIIA: one co-occurring mutation: Gly13Asp+Thr148R

Advanced Stages IIIB-IV: four co-occurring mutations: 2xGly12Val+Gln61Leu and 2x Gly12Val+Gln61His
