# Supplementary material for: Landscape of Genomic Alterations and PD-L1 Expression in Early-Stage Non-Small-Cell Lung Cancer (NSCLC)—A Single Center, Retrospective Observational Study
Source: Int J Mol Sci. 2022 Oct 19;23(20):12511. doi: 10.3390/ijms232012511 (PMC9604339; doi:10.3390/ijms232012511)
Supplement: Supplementary file 1 [file ijms-23-12511-s001.zip › Supplementary Table S3 List of fusion transcripts _ Oncomine Focus RNA Assay.pdf]

ACTG2-ALK.A2A18
AKAP9-BRAF.A8B9.COSF1013.1
B4GALT1-RAF1.B1R8
BAG4-FGFR1.B2F6
CCDC6-RET.C1R12.COSF1271
CD74-ROS1.C6R32.COSF1202
CD74-ROS1.C6R34.COSF1200
CDC27-BRAF.C16B9.1
CEP85L-ROS1.C8R36
CUX1-RET.C10R12
EGFR-EGFR.E1E8.DelPositive.1
EML1-ABL1.E17A2
EML4-ALK.E13A20.AB462411
EML4-ALK.E18A20.COSF487.1
EML4-ALK.E20A20.COSF409.1
EML4-ALK.E2A20.COSF478.1
EML4-ALK.E6A17
EML4-ALK.E6A18
EML4-ALK.E6A19.COSF1296.1
EML4-ALK.E6aA20.AB374361
ERC1_ELKS-RET.E11R12.COSF1508
ERC1-RET.E12R12
ERC1-RET.E17R12
ERC1-RET.E7R12
ERLIN2-FGFR1.E8F2
ESRP1-RAF1.E13R6.COSF826
ETV6-NTRK3.E4N15.COSF823.1
ETV6-NTRK3.E5N15.COSF571.1
EZR-ROS1.E10R34.COSF1267
FAM131B-BRAF.F2B9.COSF1189.1
FCHSD1-BRAF.F13B9.COSF404.1
FGFR2-BICC1.F17B2
FGFR2-CASP7.F17C2
FGFR2-CIT.F17C23
FGFR2-KIAA1967_CCAR2.F17C4
FGFR2-OFD1.F17O3
FGFR3-BAIAP2L1.F17B2.COSF1347
FGFR3-TACC3.F15T11
FGFR3-TACC3.F16T10.COSF1359
FGFR3-TACC3.F16T11.COSF1348
FGFR3-TACC3.F17T10.COSF1434
FGFR3-TACC3.F17T11
FGFR3-TACC3.F17T5
FGFR3-TACC3.F17T6
FGFR3-TACC3.F17T8
FGFR3-TACC3.F17T9
FKBP15-RET.F25R12
GOLGA5-RET.G7R12

GOPC-ROS1.G4R36.COSF1188
GOPC-ROS1.G8R35.COSF1139
GTF2IRD1-ALK.G7A20
HIP1-ALK.H21A20
HIP1-ALK.H28A20
HOOK3-RET.H11R12
KIAA1468-RET.K10R12
KIAA1549-BRAF.K16B10.COSF509
KIAA1549-BRAF.K17B10
KIAA1549-BRAF.K18B9
KIF5B-ALK.K15A20.COSF1381
KIF5B-ALK.K17A20.COSF1257
KIF5B-ALK.K24A20.COSF1058
KIF5B-RET.K15R12.COSF1232
KIF5B-RET.K16R12.COSF1230
KIF5B-RET.K22R12.COSF1253
KIF5B-RET.K23R12.COSF1234
KIF5B-RET.K24R11.COSF1262
KIF5B-RET.K24R8.COSF1236
KLC1-ALK.K9A20.COSF1276
LRIG3-ROS1.L16R35.COSF1269
MEMO1-ALK.M2A7
MPRIP-NTRK1.M14N12
MPRIP-NTRK1.M18N12
MPRIP-NTRK1.M21N12
NCOA4-RET.N7R12
PAPSS1-BRAF.P5B9.1
PAX8-PPARG.P10P2.COSF1219
PAX8-PPARG.P7P2.COSF1223
PAX8-PPARG.P8P2.COSF1215
PAX8-PPARG.P9P2.COSF1217
PCM1-RET.P29R12
PRKAR1A-RET.P7R12
SCAF11-PDGFRA.S1P2
SDC4-ROS1.S2R32.COSF1265
SDC4-ROS1.S2R34
SDC4-ROS1.S4R32.COSF1278
SDC4-ROS1.S4R34.COSF1280
SLC34A2-ROS1.S4R32.COSF1197
SLC34A2-ROS1.S4R34.COSF1198
SLC45A3-BRAF.S1B8.COSF871
SLC45A3-ERG.S1E4.COSF1138
SLC45A3-FGFR2.S1F1
SLC45A3-FGFR2.S1F2
SND1-BRAF.S16B9.1
SQSTM1-NTRK1.S5N10
SSBP2-NTRK1.S12N12
STRN-ALK.S3A20.COSF1430

TBL1XR1-RET.T9R12
TFG-ALK.T4A20.COSF424
TFG-ALK.T5A20.COSF426
TFG-ALK.T6A20.COSF428
TFG-NTRK1.T6N10
TMPRSS2-ERG.T1E2.COSF23.1
TMPRSS2-ERG.T1E3.COSF24
TMPRSS2-ERG.T1E4.COSF38
TMPRSS2-ERG.T1E5.COSF26
TMPRSS2-ERG.T1E6.COSF36
TMPRSS2-ERG.T1EIIIc_4
TMPRSS2-ERG.T2E2.COSF27.1
TMPRSS2-ERG.T2E4.COSF28
TMPRSS2-ERG.T2EIIIc_4
TMPRSS2-ERG.T2E5.COSF29
TMPRSS2-ERG.T3E4.COSF30
TMPRSS2-ERG.T4E4.COSF18
TMPRSS2-ERG.T4E5.COSF17
TMPRSS2-ERG.T5E4.COSF16
TMPRSS2-ERG.T5E5
TMPRSS2-ETV1.T1E5
TMPRSS2-ETV1.T2E5
TMPRSS2-ETV1.T1E4
TMPRSS2-ETV5.T1E2.EU314929
TMPRSS2-ETV5.T3E2.EU314930
TPM1-ALK.T8A20.NGS
TPM3-ALK.T7A20
TPM3-NTRK1.T7N10.COSF1318
TPR-ALK.T15A20
TPR-NTRK1.T21N10
TRIM24-BRAF.T9B9.1
TRIM27-RET.T3R12
TRIM33-RET.T16R12
VCL-ALK.V16A20.COSF1057
WIPF2-ERBB2.W1E4
HMBS.ENCTRL.E8E9
TBP.ENCTRL.E3E4
MYC.ENCTRL.E2E3
ALK.5p_NM_004304.4.e5e6
ALK.3p_NM_004304.4.e23e24
RET.5p_NM_020975.4.e6e7
RET.3p_NM_020975.4.e18e19
ROS1.5p_NM_002944.2.e11e12
ROS1.3p_NM_002944.2.e38e39
NTRK1.5p.eNST00000392302.e2e3
NTRK1.3p.eNST00000392302.e17e18
A2M-ALK.A22A19
ACBD5-RET.A11R12

AFAP1-NTRK2.A14N12
AFAP1-RET.A3R12
AGBL4-NTRK2.A6N16
AKAP13-RET.A35R12
ATIC-ALK.A7A20
BAG4-FGFR1.B1F8
BAIAP2L1-MET.B9M15
BCAN-NTRK1.B13N11
BTBD1-NTRK3.B4N14
C8orf34-MET.C2M15
CAPZA2-MET.C4M11
CARS-ALK.C17A20
CLTC-ALK.C31A20
COX5A-NTRK3.C1N15
CLTC-ROS1.C31R35
DCTN1-ALK.D26A20
EML4-ALK.E21A20
ERC1-ROS1.E11R36
ETV6-NTRK3.E4N14
ETV6-NTRK3.E5N14
FGFR1-TACC1.F17T7
FGFR2-MGEA5.F17M12
FGFR2-TACC3.F17T11
FGFR3-AES.F17A2
FGFR3-ELAVL3.F17E2
HLA_A-ROS1.H7R34
KIAA1598-ROS1.K11R36
LMNA-NTRK1.L2N11
MET-MET.M13M15
MYO5A-ROS1.M23R35
NACC2-NTRK2.N4N13
NCOA4_ELE1-RET.E6R12
NFASC-NTRK1.N20N10
OXR1-MET.O9M13
PPFIBP1-ROS1.P9R35
PTPRZ1-MET.P1M2
PTPRZ1-MET.P3M2
PTPRZ1-MET.P8M2
PWWP2A-ROS1.P1R36
QKI-NTRK2.Q6N16
RANBP2-ALK.R18A20
RNF213-NTRK1.R15N12
SEC31L1_SEC31A-ALK.S21A20
SEC31L1_SEC31A-ALK.S22A20
SPECC1L-RET.S10R12
SQSTM1-NTRK2.S5N17
TFG-MET.T5M15
TPM3-ROS1.T3R36

TPR-MET.T4M15
TRAF1-ALK.T6A20
TRIM24-NTRK2.T12N15
VCL-NTRK2.V16N12
ZCCHC8-ROS1.Z2R36
KTN1-RET.K29R12.COSF1513
TPM4-ALK.T7A20
CD74-NTRK1.C7N10
TFG-ROS1.T4R35
CD74-ROS1.C6R35
KIAA1549-BRAF.K15B9
KIAA1549-BRAF.K15B11
EZR-ROS1.E10R35
MSN-ROS1.M9R34
RUFY2-RET.R9R12
LRP1.ENCTRL.E57E58
CCDC6-ROS1.C5R35
MAGI3-AKT3.M10A3
C2orf44-ALK.C4A20
CD74-ROS1.C4R33.NGS
EML4-ALK.E13A20.COSF1062.2
EML4-ALK.E14A20.COSF1064.1
EML4-ALK.E14A20.COSF477.1
EML4-ALK.E17A20.COSF1366.2
EML4-ALK.E17A20.COSF1367.2
EML4-ALK.E20A20.COSF730.1
EML4-ALK.E2A20.COSF479.1
EML4-ALK.E6bA20.AB374362
EML4-ALK.E7A20.NGS
FGFR2-AFF3.F17A8.1
FGFR3-TACC3.F17T13.NGS
FGFR3-TACC3.F18T7.NGS
KDELR2-ROS1.K5R35
KIF5B-ALK.K15A20.COSF1060.1
KIF5B-RET.K15R11.COSF1255.1
NCOA1-ALK.N21A1.NGS
SLC34A2-ROS1.S13R32.COSF1259
SLC34A2-ROS1.S13R34.COSF1261
SLC34A2-ROS1.S13R36
SPECC1L-RET.S10R11.NGS.1
TAX1BP1-BRAF.T8B11.1
TBL1XR1-RET.T9R11.NGS.1
TMPRSS2-ETV1.T1bE4
TMPRSS2-ETV4.T1bE3
TMPRSS2-ETV5.T1bE2
TPM3-ROS1.T7R35.COSF1273
TRIM24-RET.T9R12
ITGB7.ENCTRL.E14E15

AKAP13-RET.A36R12
CCDC6-RET.C1R13
CCDC6-RET.C2R12
CCDC6-RET.C8R11
CCDC6-RET.C8R12
CLIP1-ROS1.C19R36
EML4-ALK.E3p53insA20
FGFR3-TACC3.F14T11
FGFR3-TACC3.F18T4and5
FGFR3-TACC3.F18T10.1
FGFR3-TACC3.F18T10
FGFR3-TACC3.F18T11
FGFR3-TACC3.TruncatedF17T4
SMEK2-ALK.S9A2
AGTRAP-BRAF.A5B8.COSF828.1
AXL-MBIP.A20M4.1
CEL-NTRK1.C7N7.1
EML4-ALK.E15A20.COSF413.1
IRF2BP2-NTRK1.I1N10.1
NTRK1-DYNC2H1.N17D85
PRKAR1A-ALK.P2A20.NGS.1
ALK-PTPN3.A11P3
TPR-NTRK1.T6N12.short
TPR-NTRK1.T6N12.1
EML4-ALK.E6ins18A20
EML4-ALK.E13ins90A20
EML4-ALK.E14ins2del52A20
EML4-ALK.E14ins124A20
EML4-ALK.E14del36A20
EML4-ALK.E17ins65A20
EML4-ALK.E17ins68A20
EML4-ALK.E17ins30A20_V8a
TPR-NTRK1.T21N9
CCDC6-RET.C1R12
CCDC6-RET.C1R11
CCDC6-RET.C1R11.1
FGFR3-TACC3.F17T7
FGFR3-TACC3.F17T10
FGFR3-TACC3.F17T11.1
FGFR3-TACC3.F17T11.2
CLIP4-ALK.C12A23
FGFR3-TACC3.F17intron17T4
KDELR2-ROS1.K5Rintron34
FGFR3-TACC3.F17Intron17T9
