# Supplementary material for: Landscape of Genomic Alterations and PD-L1 Expression in Early-Stage Non-Small-Cell Lung Cancer (NSCLC)—A Single Center, Retrospective Observational Study
Source: Int J Mol Sci. 2022 Oct 19;23(20):12511. doi: 10.3390/ijms232012511 (PMC9604339; doi:10.3390/ijms232012511)
Supplement: Supplementary file 1 [file ijms-23-12511-s001.zip › Supplementary Table S4 Frequency of mutations and PDL1 expression in pulmonary adenocarcinoma according to early tumour stages.pdf]

Lung Adenocarcinoma

Tumour Stage		IA1		IA2		IA3		IB		II		IIIA
Patients (n)		41		117		64		71		124		150
	n	%	n	%	n	%	n	%	n	%	n	%
EGFR	4	9.8	17	14.5	12	18.8	15	21.1	11	8.9	13	8.7
KRAS G12C	10	24.4	19	16.2	16	25.0	8	11.3	23	18.5	22	14.7
KRAS non G12C	14	34.1	19	16.2	15	23.4	15	21.1	33	26.6	35	23.3
BRAF V600E	1	2.4	6	5.1	0	0.0	0	0.0	2	1.6	1	0.7
BRAF non V600E	1	2.4	9	7.7	1	1.6	4	5.6	3	2.4	4	2.7
ERBB2	0	0	1	0.9	0	0.0	1	1.4	1	0.8	2	1.3
ALK	0	0	3	2.6	2	3.1	0	0.0	0	0.0	2	1.3
ROS1	0	0	1	0.9	0	0.0	0	0.0	0	0.0	0	0.0
RET	0	0	1	0.9	2	3.1	0	0.0	0	0.0	1	0.7
METEx14skip	0	0	3	2.6	1	1.6	2	2.8	4	3.2	3	2.0

Tumour Stage		IA1		IA2		IA3		IB		II		IIIA
Patients (n)		41		117		62		68		119		144
	n	%	n	%	n	%	n	%	n	%	n	%
PD-L1 (TPS ≥ 1)	14	34.1	46	39.3	27	43.5	31	45.6	54	45.4	69	47.9
